# Childhood Obesity Prevalence and Prevention Strategies in Primary Care: A Comprehensive Review

**DOI:** 10.7759/cureus.98456

**Published:** 2025-12-04

**Authors:** Vignesh Gunasekaran, Vishali Kapoor, Venkata Sushma Chamarthi

**Affiliations:** 1 Neonatology, West Virginia University (WVU) Medicine - Berkeley Medical Center, Martinsburg, USA; 2 Pediatrics, California Health Sciences University College of Osteopathic Medicine, Clovis, USA; 3 Pediatrics, Valley Children's Healthcare, Madera, USA

**Keywords:** behavioural interventions, body mass index: bmi, #childhood obesity, family-based therapy, prevention strategies, primary care medicine

## Abstract

Childhood obesity has become one of the most significant public health challenges of the modern era. This chronic and multifactorial condition contributes to numerous metabolic, cardiovascular, and psychosocial complications, many of which extend into adulthood. The primary care setting provides a key opportunity for early identification, prevention, and management, but remains underutilized in practice. Evidence supports routine screening from early childhood, accompanied by comprehensive behavioral interventions for children identified as overweight or obese. Effective prevention approaches in primary care include family-based behavioral strategies, motivational interviewing (MI), nutrition-focused counseling, promotion of regular physical activity, and reduction of sedentary behaviors. Multi-component interventions delivered over an extended period tend to produce the most meaningful improvements in weight-related outcomes. Despite these advances, primary care providers continue to face challenges such as time limitations, inadequate training, lack of community resources, and difficulties maintaining family engagement. Integrating team-based care, leveraging electronic health record (EHR) tools, and following structured, staged treatment models can enhance implementation. This review highlights current approaches to childhood obesity prevention and management in primary care and offers practical strategies to support clinicians in addressing this ongoing epidemic.

## Introduction and background

Childhood obesity represents one of the most significant public health crises of our generation, characterized by excessive accumulation of body fat that adversely affects a child's health and well-being [1]. Childhood obesity, defined as a child's BMI meeting or exceeding the 95th percentile on age- and sex-adjusted CDC growth reference charts, has attained epidemic status globally [2]. In the United States, approximately 14.7 million children and adolescents aged 2-19 years are affected by obesity, representing 19.7% of this population [3,4]. According to 2022 data from the WHO, the global burden includes approximately 390 million young people aged 5-19 years with overweight status, including 160 million who meet obesity criteria [5].

The consequences of childhood obesity extend far beyond childhood, with obese children having a significantly elevated risk of remaining obese into adulthood and developing serious comorbidities including type 2 diabetes mellitus, cardiovascular disease, hypertension, dyslipidemia, non-alcoholic fatty liver disease, sleep apnea, orthopedic complications, and psychosocial difficulties [6]. The economic burden is staggering, with estimated annual medical costs of childhood obesity in the United States exceeding $14 billion, and projections suggesting these costs could reach $45 billion by 2050 [7].

Primary care settings serve as the medical home for most children and represent an ideal venue for obesity prevention and early intervention [8]. Pediatric primary care providers have regular contact with families throughout childhood, providing opportunities for longitudinal monitoring, anticipatory guidance, and implementation of evidence-based interventions. In 2023, the American Academy of Pediatrics (AAP) released comprehensive clinical practice guidelines emphasizing the critical role of primary care in the evaluation and treatment of childhood obesity [9]. Similarly, the United States Preventive Services Task Force (USPSTF) recommends screening children aged 6 years and older for obesity and referring them to comprehensive, intensive behavioral interventions [10].

**Figure 1:**
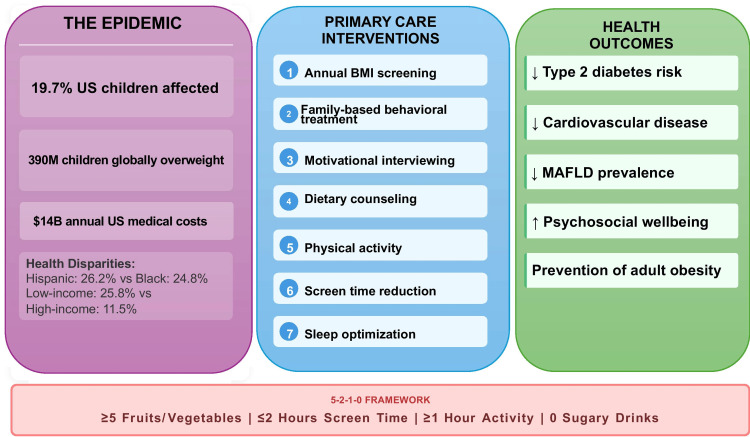
Overview of primary care-based childhood obesity prevention. This infographic illustrates evidence-based strategies within the medical home model to address the childhood obesity epidemic. The left panel highlights the burden and disparities of obesity, noting that 19.7% of U.S. children are affected, 390 million children are overweight globally, and annual U.S. medical costs exceed $14 billion. The central panel outlines primary care interventions, annual BMI screening, family-based behavioral treatment, motivational interviewing, dietary counseling, promotion of physical activity, screen time reduction, and sleep optimization. The right panel links these interventions to improved health outcomes, including reduced risks of type 2 diabetes, cardiovascular disease, and metabolic-associated fatty liver disease (MAFLD), enhanced psychosocial well-being, and prevention of adult obesity. The 5-2-1-0 framework at the bottom reinforces key daily behavioral goals: ≥5 servings of fruits/vegetables, ≤2 hours of screen time, ≥1 hour of physical activity, and 0 sugary drinks. Source: References [3, 5, 7, 9]. Figure created entirely by the authors (Chamarthi VS, Kapoor V, Gunasekaran V) in Microsoft PowerPoint using publicly available, non-copyrighted data; no permissions required.

Despite these recommendations, childhood obesity prevention and treatment remain underutilized in primary care settings, with providers citing barriers including time constraints, insufficient training, lack of effective tools, limited referral resources, and challenges with family motivation and engagement [11,12]. This review synthesizes current evidence regarding the prevalence, etiology, health consequences, and evidence-based prevention strategies for childhood obesity specifically within the primary care context, providing practical guidance for clinicians committed to addressing this epidemic (Figure 1). This narrative review emphasizes literature published from 2015 to 2025, with particular focus on recent systematic reviews, clinical practice guidelines, and evidence-based interventions from 2020 onward [3,5,9,10].

## Review

Epidemiology and prevalence

Global and United States Prevalence

Over the last four decades, childhood obesity rates have surged globally at an alarming pace. WHO data from 2022 documented that excess weight affected approximately 390 million young people between ages 5 and 19 years worldwide, with 160 million meeting obesity classification [5]. The proportion of overweight youth in this age bracket has more than doubled, escalating from 8% in 1990 to 20% by 2022, with relatively comparable rates between male and female populations. Among younger children under age 5, global overweight prevalence reached approximately 35 million in 2024 [5].

Within the United States, data spanning 2017 through March 2020 revealed that obesity affected 19.7% of the pediatric population ages 2-19 years, representing roughly 14.7 million young Americans [3]. The burden demonstrates an age-related gradient: preschool-aged children (2-5 years) showed 12.7% prevalence, elementary-aged youth (6-11 years) exhibited 20.7%, while adolescents (12-19 years) demonstrated the highest rate at 22.2% [3]. Projection models indicate a concerning trajectory, estimating that, absent meaningful intervention strategies, global obesity will affect more than 250 million children and adolescents by 2030 [13].

Racial, Ethnic, and Socioeconomic Disparities

**Figure 2:**
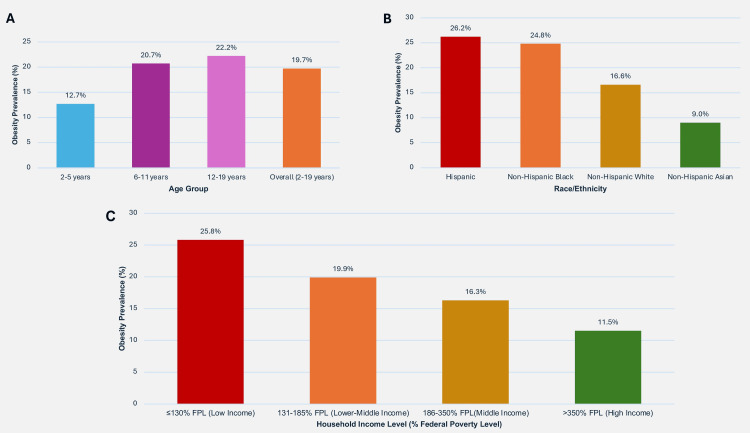
Prevalence of childhood obesity. This figure depicts childhood obesity distribution patterns among U.S. children ages 2-19 years across three demographic dimensions. Panel (A) illustrates age-based progression from 12.7% (ages 2-5 years) to 22.2% (ages 12-19 years). Panel (B) shows racial and ethnic differences, with Hispanic (26.2%) and non-Hispanic Black (24.8%) populations demonstrating higher prevalence compared to non-Hispanic White (16.6%) and non-Hispanic Asian (9.0%) groups. Panel (C) depicts the socioeconomic gradient, contrasting 25.8% prevalence in low-income families (≤130% FPL) with 11.5% in higher-income households (>350% FPL). These patterns highlight marked disparities by age, race/ethnicity, and economic status in pediatric obesity across the nation. Data source: All prevalence data in this figure are derived from the CDC National Health and Nutrition Examination Survey (NHANES), collected during 2017-March 2020, and represent the most current comprehensive national data for U.S. children and adolescents ages 2-19 years [3,14]. Figure created entirely by the authors (Chamarthi VS, Kapoor V, Gunasekaran V) in Microsoft PowerPoint using public-domain data; no permissions required. FPL: Federal Poverty Level.

Concerning disparities exist across racial, ethnic, and socioeconomic groups (Figure 2). Hispanic children demonstrate the highest prevalence at 26.2%, followed by non-Hispanic Black children at 24.8%, non-Hispanic White children at 16.6%, and non-Hispanic Asian children at 9.0% [3]. These disparities are further exacerbated by socioeconomic factors, with obesity prevalence reaching 25.8% among children from families with incomes at or below 130% of the federal poverty level, compared to 11.5% among those from families with incomes exceeding 350% of the poverty level [3]. The prevalence of severe obesity (BMI ≥120% of the 95th percentile or BMI ≥35 kg/m²) has increased disproportionately in recent years, affecting approximately 6% of children and adolescents in the United States [14].

Etiology and risk factors

Childhood obesity results from a complex interplay of genetic, biological, behavioral, environmental, and socioeconomic factors that promote positive energy balance [1,15]. Genetic factors contribute significantly to obesity risk, with heritability estimates ranging from 40% to 70% [16]. Behavioral factors represent modifiable contributors to childhood obesity, including excessive consumption of sugar-sweetened beverages, high-calorie processed foods, insufficient physical activity, and excessive sedentary behaviors, particularly screen time exceeding 2 hours per day [17,18]. Sleep duration is inversely associated with obesity risk, with inadequate sleep disrupting metabolic regulation and increasing appetite [19].

The obesogenic environment encompasses multiple influences that promote excessive energy intake and insufficient physical activity. Food insecurity paradoxically increases obesity risk through reliance on inexpensive, energy-dense, nutrient-poor foods [20]. Limited access to safe recreational spaces, walkable neighborhoods, and affordable healthy foods disproportionately affects low-income communities and communities of color [15]. Family dynamics significantly influence childhood obesity risk, with parental obesity strongly predicting childhood obesity through both genetic and environmental mechanisms. Parenting practices regarding feeding, including restrictive feeding and the use of food as a reward, can promote unhealthy eating behaviors [21].

Health consequences of childhood obesity

Metabolic and Cardiovascular Complications

Type 2 diabetes mellitus, once rare in children, has increased dramatically with rising obesity rates. Children with obesity have significantly elevated risk for insulin resistance, prediabetes, and type 2 diabetes [22]. Dyslipidemia affects 40-50% of children with obesity, while metabolic syndrome affects approximately 30% of adolescents with obesity [23]. Hypertension affects 3.8-24.8% of children with obesity, with rates considerably higher than in children with healthy weight. Left ventricular hypertrophy and increased carotid intima-media thickness, markers of early cardiovascular disease, are present in many children with obesity, predicting increased risk for premature cardiovascular disease in adulthood [5,24].

Hepatic and Pulmonary Complications

Metabolic-associated fatty liver disease (MAFLD) represents the most common liver disease in children, affecting 30-40% of children with obesity [25,26]. MAFLD can progress to non-alcoholic steatohepatitis, cirrhosis, and hepatocellular carcinoma [27]. Obstructive sleep apnea affects 10-60% of children with obesity, depending on severity, contributing to metabolic dysfunction, behavioral problems, and impaired academic performance [28,29]. Asthma prevalence is significantly elevated in children with obesity, with obesity contributing to asthma severity and treatment resistance [5,24,30].

Psychosocial Consequences and Persistence into Adulthood

Children with obesity experience significantly elevated rates of depression, anxiety, low self-esteem, and poor body image. Weight-based teasing and bullying are pervasive, with adverse effects on mental health, social functioning, and academic performance. Quality of life scores in children with severe obesity are comparable to those of children with cancer [31]. Obesity during childhood and adolescence strongly predicts obesity in adulthood, with approximately 55% of obese children becoming obese adolescents and 80% of obese adolescents remaining obese as adults. Adult obesity significantly increases lifetime risk for cardiovascular disease, type 2 diabetes, certain cancers, and premature mortality [32].

**Figure 3:**
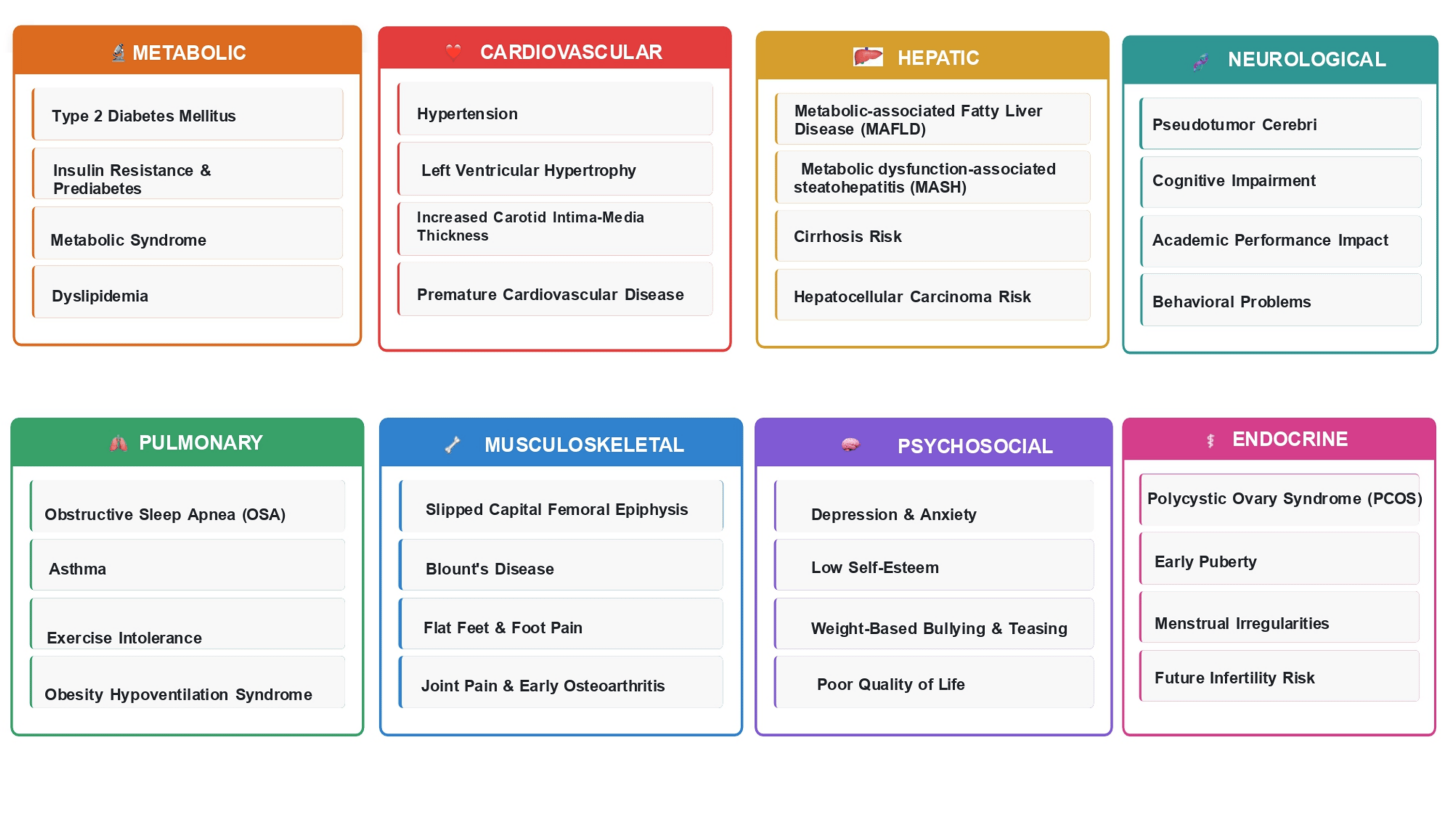
Health impacts of childhood obesity across organ systems. This figure summarizes the multisystem consequences of childhood obesity, highlighting its broad clinical impact beyond weight status. Metabolic effects include type 2 diabetes mellitus, insulin resistance, metabolic syndrome, and dyslipidemia. Cardiovascular complications encompass hypertension, left ventricular hypertrophy, increased carotid intima-media thickness, and premature cardiovascular disease. Hepatic involvement includes metabolic-associated fatty liver disease (MAFLD), metabolic dysfunction-associated steatohepatitis (MASH), cirrhosis risk, and hepatocellular carcinoma risk. Neurological manifestations include pseudotumor cerebri, cognitive impairment, academic difficulties, and behavioral problems. Pulmonary complications include obstructive sleep apnea (OSA), asthma, exercise intolerance, and obesity hypoventilation syndrome. Musculoskeletal issues, such as slipped capital femoral epiphysis, Blount’s disease, foot deformities, and early osteoarthritis, are common. Psychosocial impacts include depression, anxiety, low self-esteem, bullying, and reduced quality of life. Endocrine disturbances include polycystic ovary syndrome (PCOS), early puberty, menstrual irregularities, and potential future infertility risk. Together, these illustrate obesity’s pervasive impact on pediatric physical and mental health [6,23-32]. Figure created entirely by the authors (Chamarthi VS, Kapoor V, Gunasekaran V) in Microsoft PowerPoint using summarized information from peer-reviewed literature; no copyrighted or adapted material included, and no permissions required.

Childhood obesity has immediate and long-term health consequences affecting multiple organ systems and psychosocial well-being (Figure 3).

Screening and assessment in primary care

BMI Screening

The AAP and USPSTF recommend annual BMI screening for all children beginning at age 2 years [9,10]. Using CDC growth charts based on age and sex, children are categorized as underweight when BMI percentiles fall below the 5th percentile, healthy weight between the 5th and 85th percentiles, overweight from the 85th to just below the 95th percentile, obesity at or above the 95th percentile, and severe obesity when BMI reaches 120% of the 95th percentile or ≥35 kg/m². While BMI has limitations and does not directly measure adiposity, it remains the most practical and validated screening tool for primary care. BMI should be plotted on growth charts at every well-child visit and during acute care visits when possible to track trajectories over time [33].

Comprehensive Assessment

For children identified with overweight or obesity, comprehensive assessment should include a detailed history addressing dietary intake patterns, physical activity levels, sedentary behaviors, sleep duration and quality, and psychosocial factors including mood and self-esteem [9]. Family history of obesity and cardiometabolic diseases should be documented. Physical examination should assess for acanthosis nigricans (a marker of insulin resistance), hepatomegaly, blood pressure elevation, and orthopedic abnormalities. The AAP recommends laboratory screening for children aged 10 years and older with obesity, including a fasting lipid panel, fasting glucose or hemoglobin A1c, and alanine aminotransferase (ALT) to assess for dyslipidemia, prediabetes/diabetes, and MAFLD, respectively [9,24].

Evidence-based prevention strategies in primary care

Universal Prevention Approaches

Universal prevention strategies should be implemented for all children regardless of weight status, promoting healthy growth and preventing excessive weight gain. Anticipatory guidance at well-child visits should address key obesity-related behaviors at developmentally appropriate stages, including exclusive breastfeeding for 6 months when possible, appropriate introduction of complementary foods, healthy beverage choices (water and milk, avoiding sugar-sweetened beverages), promoting fruits and vegetables, limiting processed foods, encouraging regular family meals, promoting adequate physical activity, limiting screen time, and ensuring sufficient sleep.

The 5-2-1-0 framework provides a simple, evidence-based message for families: 5 or more servings of fruits and vegetables daily, 2 hours or less of recreational screen time daily, 1 hour or more of physical activity daily, and 0 sugar-sweetened beverages [34]. This framework has been widely adopted and can be easily reinforced in primary care visits.

Behavioral Counseling and Motivational Interviewing

Motivational interviewing (MI) represents an evidence-based, patient-centered counseling approach that enhances intrinsic motivation for behavior change [35]. MI techniques include open-ended questions, reflective listening, affirming strengths, and collaborative goal-setting. Meta-analyses demonstrate that MI interventions produce small to moderate effect sizes for improving dietary behaviors and physical activity in children [36]. Primary care providers can implement brief MI techniques during routine visits by assessing readiness for change, exploring ambivalence about behavior change, eliciting change talk through open-ended questions, developing specific realistic family-centered goals, and providing supportive follow-up. Even brief MI interventions (3-4 sessions) can produce meaningful improvements in obesity-related behaviors [23,24,37].

Family-Based Behavioral Treatment

Family-based behavioral treatment (FBT) represents the most extensively studied and effective approach for childhood obesity treatment [38]. FBT involves the entire family unit, targeting dietary modification, increased physical activity, decreased sedentary behaviors, and behavioral strategies including self-monitoring, goal-setting, stimulus control, and problem-solving. Parents serve as agents of change, modeling healthy behaviors and restructuring the home environment.

The USPSTF recommends comprehensive, intensive behavioral interventions comprising at least 26 contact hours over 2-12 months [10]. A recent randomized controlled trial demonstrated that FBT implemented in pediatric primary care produced significant improvements in child BMI, with benefits extending to parents and siblings [38]. Key components of effective FBT programs include regular contact with trained interventionists; a family-centered approach involving parents and children; behavioral self-monitoring (food logs, activity tracking); structured goal-setting with gradual sustainable changes; positive reinforcement and problem-solving; and a dual focus on dietary and physical activity changes.

Dietary and Physical Activity Interventions

Evidence-based dietary strategies include increasing consumption of fruits and vegetables, limiting sugar-sweetened beverages and juice, reducing intake of high-calorie processed foods, promoting appropriate portion sizes, encouraging regular meals including breakfast, and increasing family meal frequency [17]. Physical activity recommendations include at least 60 minutes daily of moderate-to-vigorous physical activity for children and adolescents [39]. Primary care providers can promote physical activity by assessing current activity levels, collaborating with families to identify enjoyable activities, addressing barriers to activity, and providing community resources for physical activity programs.

Reducing sedentary behaviors, particularly screen time, is associated with improved weight outcomes. Recommendations include limiting recreational screen time to less than 2 hours daily, removing televisions and electronic devices from bedrooms, establishing screen-free meal times and family times, and promoting active alternatives to screen time [18]. Adequate sleep duration is protective against obesity, with recommendations varying by age: 10-13 hours for children aged 3-5 years, 9-12 hours for children aged 6-12 years, and 8-10 hours for adolescents aged 13-18 years [19,40].

Staged Treatment Approach

**Figure 4:**
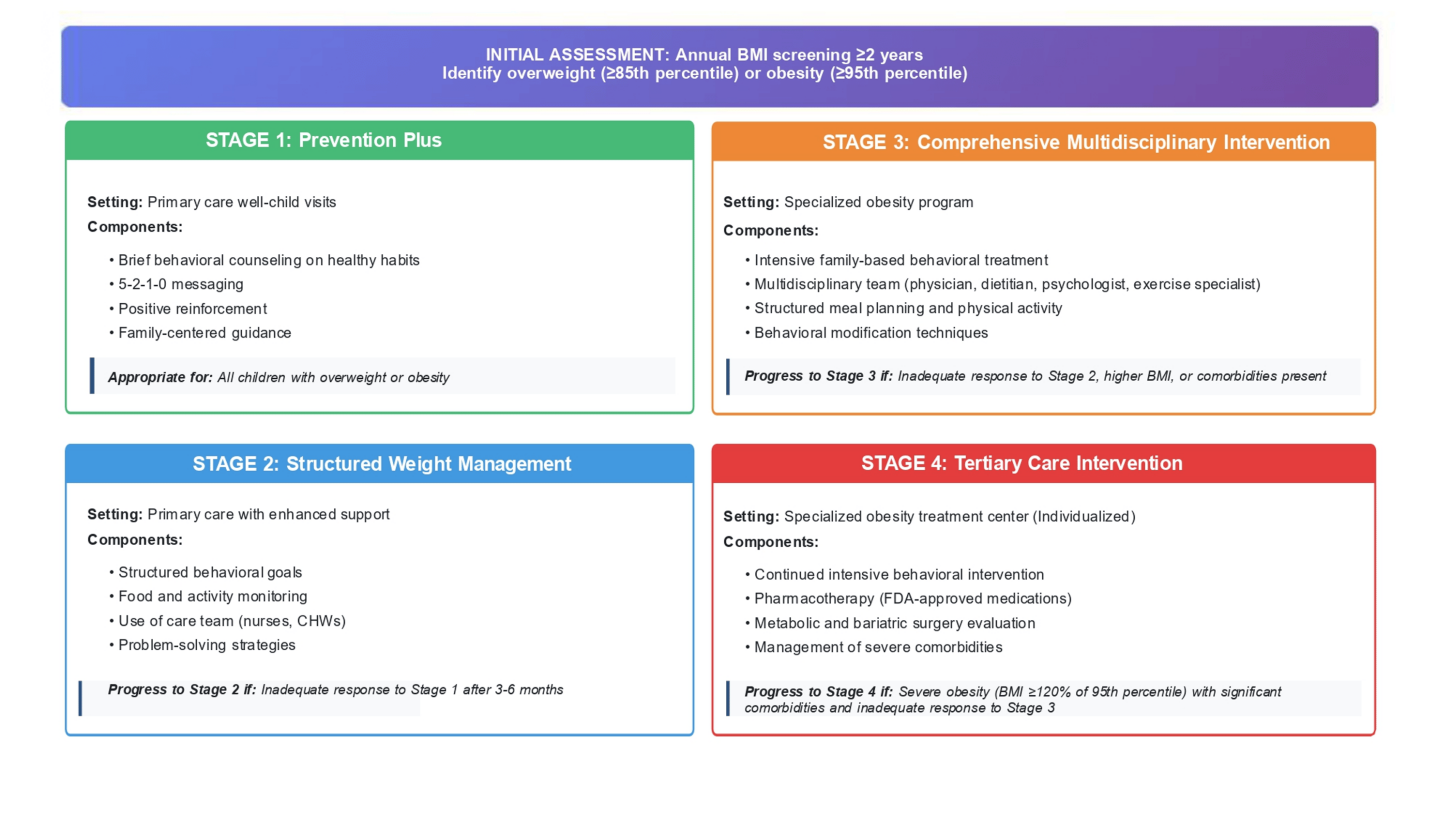
American Academy of Pediatrics (AAP) staged treatment model for childhood obesity. This figure outlines the AAP’s four-stage, evidence-based approach to managing childhood overweight and obesity within the medical home. The model begins with Initial Assessment, recommending annual BMI screening from age ≥2 years to identify overweight (≥85th percentile) or obesity (≥95th percentile). Stage 1: Prevention Plus emphasizes brief counseling during primary care visits, focusing on healthy behaviors, 5-2-1-0 messaging, positive reinforcement, and family-centered guidance. Stage 2: Structured Weight Management introduces enhanced primary care support with structured behavioral goals, food and activity monitoring, and teamwork with nurses or community health workers (CHWs). Stage 3: Comprehensive Multidisciplinary Intervention involves referral to specialized obesity programs for intensive family-based treatment led by a multidisciplinary team (physician, dietitian, psychologist, exercise specialist) using structured plans and behavioral techniques. Stage 4: Tertiary Care Intervention applies to adolescents with severe obesity (BMI ≥120% of the 95th percentile) or major comorbidities, incorporating pharmacotherapy, metabolic/bariatric surgery evaluation, and advanced behavioral management. Progression through stages depends on response, BMI severity, and comorbidities, supporting individualized, escalating care for effective long-term outcomes [41-43]. Figure created entirely by the authors (Chamarthi VS, Kapoor V, Gunasekaran V) in Microsoft PowerPoint as an original schematic based on publicly available AAP 2023 guideline concepts; no copyrighted or adapted material included, and no permissions required.

The AAP recommends a staged approach to obesity treatment, escalating intensity based on patient characteristics, response to intervention, and resource availability (Figure 4) [8,41,42]. The model begins with routine BMI screening and progresses through four treatment stages, ranging from brief counseling in primary care to comprehensive multidisciplinary and tertiary-level interventions for adolescents with severe obesity. Detailed descriptions of each stage are provided in Figure 4.

Multi-Level Interventions

Integration of community health workers (CHWs) into primary care obesity interventions shows promise for improving engagement, particularly in underserved populations [43]. CHWs provide culturally sensitive support, connect families to community resources, reinforce clinical messaging, and facilitate behavior change through home visits or group sessions [43-45]. Electronic health record (EHR) modifications, including BMI auto-calculation and percentile display, clinical decision support alerts, and standardized templates for obesity visits, improve screening rates and the quality of obesity care.

Table 1 outlines key strategies for childhood obesity prevention in primary care settings, detailing their implementation approaches, supporting evidence levels, and major recommendations for clinical practice.

**Table 1:** Evidence-based strategies for childhood obesity prevention and management in primary care. This table outlines evidence-based strategies for obesity prevention and early intervention within the pediatric medical home. The Category column organizes interventions into seven key domains: screening and assessment, universal prevention, behavioral interventions, dietary interventions, physical activity and sedentary behavior, staged treatment approach, and system-level strategies. It summarizes approaches across screening, behavioral, dietary, physical activity, sleep interventions, staged treatment, and system-level recommendations. Evidence levels are categorized as Strong (supported by multiple high-quality randomized controlled trials or systematic reviews/meta-analyses), Moderate (supported by promising preliminary data), and Emerging (supported by limited evidence). Data sources: American Academy of Pediatrics Clinical Practice Guideline for Childhood Obesity (2023) [9]; U.S. Preventive Services Task Force (USPSTF) recommendations (2024) [10]; and supporting evidence from cited references. Table created entirely by the authors (Chamarthi VS, Kapoor V, Gunasekaran V) in Microsoft Word using publicly available guideline content; no copyrighted or adapted material included, and no permissions required.

Category	Strategy	Implementation in Primary Care	Evidence Level	Key Recommendations
Screening and Assessment	Annual BMI Screening	- Calculate BMI percentile at every well-child visit (≥2 years) - Use EHR auto-calculation tools - Screen during acute visits when possible	Strong	- Identify overweight (≥85th percentile) and obesity (≥95th percentile) early - Track BMI trajectory over time
	Comprehensive Assessment	- Dietary intake patterns and beverage consumption - Physical activity and sedentary behavior assessment - Sleep duration and quality - Psychosocial screening (mood, self-esteem) - Family history of obesity and cardiometabolic disease	Strong	- Physical exam: acanthosis nigricans, hepatomegaly, BP - Lab screening (≥10 years with obesity): lipids, glucose/HbA1c, ALT - Assess for comorbidities
Universal Prevention (All Children)	5-2-1-0 Framework	- 5 or more servings of fruits and vegetables daily - 2 hours or less of recreational screen time daily - 1 hour or more of physical activity daily - 0 sugar-sweetened beverages	Strong	- Simple, memorable message for families - Incorporate into anticipatory guidance - Provide printed materials - Widely adopted evidence-based framework
	Anticipatory Guidance	- Age-appropriate obesity prevention counseling at all visits - Breastfeeding support (exclusive 6 months when possible) - Healthy complementary food introduction - Address sleep hygiene	Moderate	- Tailor messages to developmental stage - Focus on health rather than weight - Provide positive, actionable guidance
Behavioral Interventions	Family-Based Behavioral Treatment (FBT)	- Involve entire family unit - Include behavioral strategies: self-monitoring, goal setting, stimulus control - Regular contact with trained interventionists	Strong	- Parents as agents of change - Restructure home environment - Benefits extend to siblings
	Motivational Interviewing (MI)	- Use open-ended questions - Reflective listening - Affirm strengths and efforts - Collaborative goal-setting - Assess readiness for change	Moderate	- Enhances intrinsic motivation - Brief interventions (3-4 sessions) are effective - Explore ambivalence non-judgmentally - Small to moderate effect sizes - Can be integrated into routine visits
Dietary Interventions	Nutritional Counseling	- Increase fruits and vegetables (≥5 servings/day) - Eliminate/limit sugar-sweetened beverages and juice - Reduce high-calorie processed foods - Promote appropriate portion sizes - Encourage regular meals including breakfast - Increase family meal frequency	Strong	- Provide specific, actionable recommendations - Focus on adding healthy foods, not just restriction - Consider cultural food preferences - Referral to registered dietitian when available
	Sugar-Sweetened Beverage Reduction	- Eliminate sodas, sports drinks, sweetened teas - Limit 100% fruit juice to 4-6 oz/day (if any) - Promote water as primary beverage - Low-fat/non-fat milk appropriate for age	Strong	- Single most effective dietary change - Significant reduction in caloric intake - Address parental misconceptions about juice
Physical Activity and Sedentary Behavior	Physical Activity Promotion	- Assess current activity levels - Collaborate to identify enjoyable activities - Address barriers (safety, cost, time) - Provide community resource list - Encourage active transportation (walking, biking) - Promote unstructured active play	Strong	- Goal: ≥60 minutes/day MVPA - Can be accumulated throughout day - Family participation increases success - Focus on fun, not performance
	Screen Time Reduction	- Limit recreational screen time to <2 hours/day - Remove TVs/devices from bedrooms - Establish screen-free mealtimes - Promote active alternatives	Strong	- Strongly associated with obesity risk - Reduce sedentary behavior - Decrease exposure to food marketing - Create family media use plan
	Sleep Optimization	- Assess sleep duration and quality - Establish consistent bedtime routines - Screen-free bedrooms - Address sleep disorders (OSA screening)	Moderate	- Age-appropriate recommendations: ◦ 3-5 years: 10-13 hours ◦ 6-12 years: 9-12 hours ◦ 13-18 years: 8-10 hours - Inadequate sleep increases obesity risk
Staged Treatment Approach	Stage 1: Prevention Plus	- Brief counseling in primary care (5-10 min) - Focus on healthy behaviors - Implement at routine visits - 5-2-1-0 messaging	Moderate	- Appropriate for all children with overweight/obesity - Low intensity, easy to implement - First step in treatment algorithm
	Stage 2: Structured Weight Management	- Monthly follow-up visits - Structured behavioral goals - Systematic monitoring (food logs, activity)	Moderate	- For children not responding to Stage 1 - Requires more provider time - Can utilize care team (nurses, CHWs)
	Stage 3: Comprehensive Multidisciplinary	- Referral to structured programs - Frequent contact (weekly sessions) - Multidisciplinary team: physicians, RDs, mental health, exercise specialists	Strong	- For children with inadequate response to Stages 1-2 - Higher BMI or comorbidities - Requires specialized resources
	Stage 4: Tertiary Care	- Referral to specialized obesity centers - Consideration of pharmacotherapy - Metabolic and bariatric surgery evaluation - For severe obesity with comorbidities	Strong	- Adolescents with severe obesity (BMI ≥120% of 95th percentile) - Significant comorbidities - Failed response to Stage 3
System-Level Strategies	Community Health Workers (CHWs)	- Integration into primary care team - Culturally sensitive support - Connect families to community resources - Home visits or group sessions - Reinforce clinical messaging	Emerging	- Improves engagement in underserved populations - Addresses social determinants of health - Facilitates behavior change - Cost-effective intervention extender
	EHR Optimization	- Clinical decision support alerts - Standardized templates for obesity visits - Tracking and reporting tools	Moderate	- Improves screening rates and quality of obesity care - Facilitates population health management - Supports quality improvement initiatives
	Provider Training	- Continuing education in MI techniques - Behavioral counseling skills - Obesity management guidelines - Communication skills (non-stigmatizing language) - Cultural competency	Moderate	- Increases provider confidence - Improves quality of counseling - Reduces treatment barriers - Essential for practice change

Barriers and challenges in primary care

Despite strong evidence supporting primary care-based obesity prevention, multiple barriers impede implementation. Primary care providers report insufficient time during visits to address obesity comprehensively, with competing priorities for preventive care, acute concerns, and chronic disease management [11,46]. Many providers lack confidence in obesity counseling due to insufficient training in behavior change techniques, MI, and obesity management. Concerns about damaging the therapeutic relationship, causing emotional harm, or appearing judgmental contribute to reluctance in addressing weight directly [12].

Many parents inaccurately perceive their child's weight status, particularly among younger children and boys, resulting in low motivation for behavior change. Families face substantial barriers including limited financial resources for healthy foods and physical activity programs, time constraints related to work schedules and competing family demands, lack of access to safe recreational spaces in neighborhoods, limited availability of healthy food options in food deserts, and cultural beliefs about appropriate child weight and feeding practices [11,12].

Healthcare systems often lack adequate reimbursement for obesity counseling and intervention, limiting the time providers can dedicate to this complex issue. Insufficient referral resources for intensive behavioral interventions, registered dietitian services, and mental health support constrain treatment options. Fragmented care between primary care, specialty services, and community programs further impedes comprehensive management.

## Conclusions

Childhood obesity affects nearly one in five children in the United States, establishing trajectories toward chronic disease and premature mortality that extend far beyond childhood. Primary care settings offer unique opportunities for early identification and intervention through systematic BMI screening beginning at age 2 years, coupled with comprehensive behavioral interventions for children with overweight or obesity. Evidence strongly supports family-centered behavioral treatment delivered with adequate intensity (at least 26 contact hours), incorporating motivational interviewing techniques, promotion of healthy dietary patterns, encouragement of at least 60 minutes of daily physical activity, reduction of screen time to less than 2 hours daily, and optimization of age-appropriate sleep duration. Multi-component interventions addressing multiple behaviors simultaneously demonstrate greater efficacy than single-component approaches, with staged treatment algorithms providing a practical framework for escalating care from universal prevention to intensive multidisciplinary interventions when needed.

Translating evidence into practice requires addressing substantial barriers through enhanced provider training in behavioral counseling, integration of multidisciplinary team members to increase intervention intensity, utilization of EHR support tools, development of structured referral pathways, improved reimbursement policies, and community partnerships addressing social determinants of health. While reversing the obesity epidemic requires coordinated efforts across multiple sectors, including public policy, food systems, schools, and healthcare systems, primary care providers occupy a pivotal position to identify at-risk children early, deliver evidence-based interventions, and coordinate comprehensive care. By implementing the strategies outlined in this review, primary care clinicians can make meaningful contributions to reversing the childhood obesity epidemic and improving lifelong health trajectories, making this effort among the most important priorities in contemporary pediatric primary care.
